# Low and high stimulation frequencies differentially affect automated response selection in the superior parietal cortex – implications for somatosensory area processes

**DOI:** 10.1038/s41598-020-61025-y

**Published:** 2020-03-03

**Authors:** Julia Friedrich, Christian Beste

**Affiliations:** 1 Cognitive Neurophysiology, Department of Child and Adolescent Psychiatry, Faculty of Medicine, TU Dresden, Germany; 2 Faculty of Psychology, School of Science, TU Dresden, Germany

**Keywords:** Cognitive control, Perception

## Abstract

Response inhibition as a central facet of executive functioning is no homogeneous construct. Interference inhibition constitutes a subcomponent of response inhibition and refers to inhibitory control over responses that are automatically triggered by irrelevant stimulus dimensions as measured by the Simon task. While there is evidence that the area-specific modulation of tactile information affects the act of action withholding, effects in the context of interference inhibition remain elusive. We conducted a tactile version of the Simon task with stimuli designed to be predominantly processed in the primary (40 Hz) or secondary (150 Hz) somatosensory cortex. On the basis of EEG recordings, we performed signal decomposition and source localization. Behavioral results reveal that response execution is more efficient when sensory information is mainly processed via SII, compared to SI sensory areas during non-conflicting trials. When accounting for intermingled coding levels by temporally decomposing EEG data, the results show that experimental variations depending on sensory area-specific processing differences specifically affect motor and not sensory processes. Modulations of motor-related processes are linked to activation differences in the superior parietal cortex (BA7). It is concluded that the SII cortical area supporting cognitive preprocessing of tactile input fosters automatic tactile information processing by facilitating stimulus-response mapping in posterior parietal regions.

## Introduction

Inhibition of actions or thoughts plays a key role in everyday life since it is necessary to maintain goal-directed behavior. Since visual or auditory input is not always available to inform behavior, it is often also important to rely on tactile information. Commonly, it is not only necessary to completely withhold an action, but it is more essential to inhibit distraction in the first place to accomplish efficient action control. It is known that inhibitory control as a central executive function^1,2^ is not a homogeneous construct^1^. Rather it consists of subcomponents^3,4^ like the ‘inhibition of interferences’ and ‘action inhibition’ that need to be separately addressed in order to fully understand response inhibition processes. It has recently been shown that tactile information efficiently trigger response inhibition^5^ and that the variation of the area-specificity of tactile input has differential effects on the efficacy of inhibitory control processes^6^. Yet, previous work has primarily focused on action inhibition and peculiarities of the somatosensory system for other subcomponents of inhibitory control and the associated neurophysiological mechanisms  remain  largely elusive.

Interference inhibition, as another component of response inhibition, involves inhibitory control over responses that are automatically triggered by irrelevant stimulus dimensions and can be measured using a Simon task^3^. The Simon task constitutes a standard paradigm in the context of inhibitory control^1^. In a typical Simon task, one stimulus is assigned to a key press on the left and another stimulus to a key press on the right. After that assignment, one stimulus at once is presented either on the left or the right^1,7,8^. In other words, stimulus presentation is lateralized, whereas a non-spatial stimulus feature requires a lateralized response^9^. Although stimulus location is not relevant, it still interferes with the correct response, which was assigned to the stimulus at the beginning of the experiment^1^. Correspondence between the response location associated with the task-relevant stimulus feature and the task-irrelevant location of the presented stimulus (i.e. congruent trials) results in better performance than non-correspondence (i.e. incongruent trials)^8,10^. Also in tactile versions of the Simon task using low and high intensities^11,12^ or continuous and pulsed stimulation^13^ as relevant stimulus features, this “Simon effect” can be found^8^. Processes occurring in the Simon task are often described by dual-process models^8,13–15^, which assume an occurrence of automatic and controlled processes^10,16,17^. According to these models, an “unconditionally automatic” (task-irrelevant) process, also called “direct route”, is triggered by stimuli in a way that facilitates responses directly corresponding to their location. A second, rather controlled process, running in parallel, constitutes deliberate/controlled response selection based on conditions defined by the task-relevant stimulus-response assignment and is also referred to as the “indirect route”^8,13,18,19^. Hence, in case of incongruency, the task-irrelevant stimulus feature automatically activating the response towards stimulus location is in conflict with actual task-relevant demands. Resolving this conflict usually requires cognitive control^13,20^.

If conflicts impair the process of response selection^20^, the question arises whether somatosensory cortices differ regarding the modulation of response selection/preparation processes during different processing modes (i.e. automatic and controlled). This question is of importance because the primary (SI) as well as the secondary somatosensory cortex (SII)  show different neuroanatomical connections to motor areas^21^, as well as to the inferior and orbital frontal cortices^21–24^ known to play an important role in the cortical (response) inhibition network^25^. Even more important, the primary (SI) as well as the secondary somatosensory cortex (SII) is activated by tactile input, yet only the SII has been associated with cognitive aspects of tactile information processing like comparing present and past stimulus input relevant for behavioral decisions^26,27^. This suggests a “cognitive pre-processing” function of the SII. On this basis, we assume that either in incongruent trials when a response conflict occurs, SII-triggered processes result in superior performance than SI-triggered processes because cognitive pre-processing of information facilitates response selection/preparation. Yet, it is also plausible to assume that area-specific effects occur in congruent trials since no cognitive control has to be engaged when no conflict is arising. Therefore, cognitive pre-processing may be rapidly completed, which may result in more efficient performance. The experimental design makes use of the fact that SI and SII predominantly respond to different frequencies ranges, which are known as flutter and vibration^28^. On the individual subject level, the SI has been found to be more often activated by a 35 Hz frequency as compared to the SII and the opposite was shown for the 150 Hz stimulation^29^. Another fMRI study investigated the frequency range from 20 to 200 Hz^30^. SI activation was stronger during 40 Hz as compared to 80 Hz stimulation and activation declined even further at higher frequencies. The SII, however, was active in response to flutter and vibration frequencies. Although results with regard to frequency-specificity do not seem completely straightforward, SII activity is still predominantly triggered by Pacinian mechanoreceptive afferents^28,29,31^, which respond to higher (vibration) frequencies^32^. We are therefore convinced that our assumptions that 40 Hz stimulation triggers predominant SI activation and 150 Hz SII activation are sufficiently justified even though we did not measure SI or SII activation directly. Recent work already showed that SI and SII areas differ in their efficacy to trigger sensorimotor integration to affect motor control^6^.

To address the question whether somatosensory cortices differ regarding the modulation of response selection/preparation processes during different processing modes, we conducted electrophysiological recordings (EEG) and combined it with source localization analyses (sLORETA)^33^ and temporal decomposition methods (RIDE)^34^ to examine underlying neurophysiological processes and functional neuroanatomical areas associated with them. Since the Simon task provides a measure of stimulus-response conflict^7,9,18,35,36^, event-related potential (ERP) components modulated by conflict like the N2 are examined^37,38^. Simon conflicts have been associated with an increased (more negative) amplitude of the N2 ERP-component, which has been linked to activation modulations in the anterior cingulate cortex (ACC)^18,38–47^. Importantly, however, it has to be noted that that ERP-components, like the N2, are composed of various signals and information from different sources^48–51^. Especially the N2 ERP-component has been shown to reflect a mixture of stimulus-related perceptual processes and response-related motor processes in the context of conflict monitoring^37,52,53^. To account for this problem, we conducted residue iteration decomposition (RIDE)^54^. This method allows the temporal decomposition of the EEG signal resulting in clusters with different functional relevance. The S-cluster is assumed to comprise stimulus-related codes (i.e. perception and attention) whereas the R-cluster contains response-related codes like response preparation or execution^34,54,55^. The C-cluster reflects intermediate processes between the S- and R-cluster like response selection^54,56–58^. Based on recent results demonstrating that the modulation of conflict can be reflected in the S- and the R-cluster^52^, both clusters were quantified. Also, the C-cluster was quantified since the modulation of tactile information has already been linked to response selection processes when measuring inhibitory control by means of a Go/Nogo task^6,59^. However, it can be assumed that the process of response inhibition (i.e. a complete inhibition of the motor response) provoked by a Go/Nogo paradigm is modulated differently as compared to the stimulus-response conflict evoking the Simon effect (i.e. integration of task-relevant and –irrelevant stimulus features)^35^. We hypothesize that the R-cluster is modulated since it has already been linked to conflict and the inhibition of interference^52^. Regarding underlying functional neuroanatomical structures, it should be noted that the somatosensory system projects information to several other areas like the posterior parietal and the premotor cortex but also the prefrontal cortex^26,27,60,61^. Yet, since the posterior parietal cortex has been associated with the transformation of tactile input into external space^62^, which is an essential prerequisite for the Simon task, it seems plausible that it reflects area-specific effects on response selection/preparation during different processing modes. Furthermore, the posterior parietal cortex is assumed to play a major role in the integration of touch information and action^26,60,62^ and the superior parietal cortex has already been linked to tactile object localization^63^. Importantly, this area also codes intended movement goals^64,65^. We assume that SII-triggered processes are associated with stronger posterior parietal cortex activation reflecting stronger stimulus-response mapping since the cognitive preprocessing function of this area might lead to more efficient sensorimotor integration.

## Results

### Behavioral data

The repeated measures ANOVA calculated for the percentage of hits revealed a main effect of “congruency” [F(1,22) = 37.53; p < 0.001; *η*_*p*_^*2*^ = 0.630]. More hits occurred in congruent (94% ± 1.2) than in incongruent trials (89% ± 1.7). Furthermore, the interaction of “congruency x frequency” [F(1,22) = 6.3; p = 0.020; *η*_*p*_^*2*^ = 0.223] was significant. Post-hoc paired tests showed that 40 and 150 Hz conditions did not differ in the congruent [t(22) = −0.62; p = 0.544], but in the incongruent condition [t(22) = 2.24; p = 0.035]. In incongruent trials, 40 Hz stimulation resulted in more hits (89% ± 1.9) than 150 Hz stimulation (88% ± 1.7). The main effect of “frequency” was not significant [F(1,22) = 1.01; p = 0.326; *η*_*p*_^*2*^ = 0.044].

Regarding reaction times (RTs), the repeated measures ANOVA showed a main effect of “congruency” [F(1,22) = 225.45; p < 0.001; *η*_*p*_^*2*^ = 0.911]. Reactions were faster in congruent (477 ms ± 14) than in incongruent trials (521 ms ± 13). Besides that, the interaction of “congruency x frequency” [F(1,22) = 9.28; p = 0.006; *η*_*p*_^*2*^ = 0.297] was significant. Post-hoc paired t-tests revealed that 40 and 150 Hz conditions were significantly different in congruent [t(22) = 2.58; p = 0.017] but not in incongruent trials [t(22) = 0.13; p = 0.900]. In congruent trials, the 150 Hz stimulation produced faster reaction times (470 ms ± 15) than the 40 Hz stimulation (483 ms ± 13). In incongruent trials, 40 Hz (522 ms ± 12) and 150 Hz conditions (521 ms ± 14) produced almost identical reaction times. The main effect of “frequency” was not significant [F(1,22) = 2.25; p = 0.148; *η*_*p*_^*2*^ = 0.093]. Details can be found in Table 1.

**Table 1:** Behavioral data for different congruency and frequency conditions.

congruency	frequency	*hit rates*	*hit reaction times*
congruent	40 Hz	94%	484 ms
	150 Hz	94%	470 ms
incongruent	40 Hz	90%	522 ms
	150 Hz	88%	521 ms

Interaction effects. Mean hit rates and hit reaction times.

The above described analyses revealed differential effects of the predominant processing area and experimental condition (congruent vs. incongruent), depending on whether the RT data or the accuracy data was analyzed. To account for these aspects, we calculated an “inverse efficiency index (IES)”. For this purpose, the ratio of RT and accuracy was  computed. The smaller the IES, the better the performance. The analysis revealed a main effect of “congruency” [F(1,22) = 111.04; p < 0.001; *η*_*p*_^*2*^ = 0.835] with superior performance in the congruent condition. Additionally, the interaction of “congruency x frequency” [F(1,22) = 12.95; p = 0.002; *η*_*p*_^*2*^ = 0.370] was significant. Post-hoc paired t-tests showed that 40 and 150 Hz conditions were not significantly different in incongruent trials [t(22) = −1.04; p = 0.312]. Yet, in congruent trials, the 150 Hz stimulation resulted in superior performance as compared to the 40 Hz condition [t(22) = 2.28; p = 0.033]. To summarize, the behavioral data show that only performance in congruent, but not in incongruent trials was differentially affected by the cortical area predominantly processing sensory input in the experiment.

### Standard event-related potentials (ERP components)

**Figure 1:**
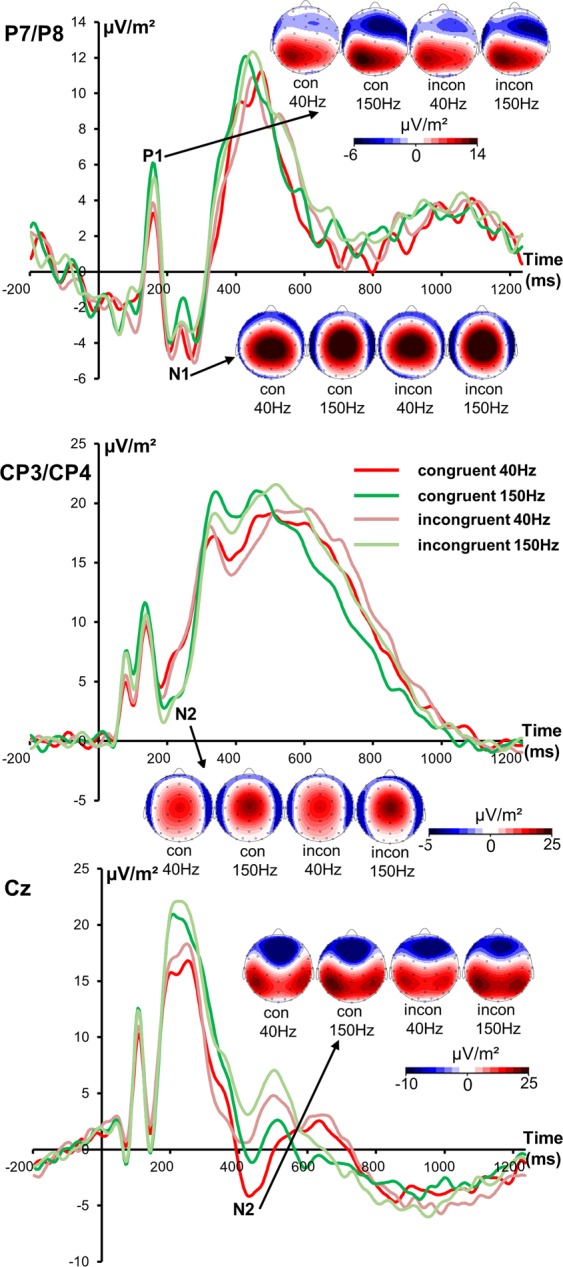
The P1 and N1 ERP-components averaged across electrodes P7 and P8 (upper part of the figure) as well as the N2 component averaged across electrodes CP3 and CP4 (middle part of the figure) and the N2 component at electrode Cz (lower part of the figure) in 40 and 150 Hz congruent and incongruent trials. Different colors of the ERPs reflect different conditions (40 and 150 Hz congruent or incongruent trials) as can be seen in the legend. Time point 0 represents stimulus presentation in all plots. The scalp topography plots represent the P1, N1 and N2 under different conditions with red indicating positive and blue negative values.

The P1, N1 and N2 ERP-components are illustrated in Fig. 1.

The repeated measures ANOVA of P1 ERP-component showed a significant main effect of “frequency” [F(1,22) = 4.46; p = 0.046; *η*_*p*_^*2*^ = 0.169]. The P1 amplitude was larger in the 150 Hz (3.8 µV/m² ± 0.8) than in the 40 Hz condition (2.6 µV/m² ± 0.71). No further main or interaction effect was significant (all F ≤ 2.02; p ≥ 0.169). For the N1 ERP-component, no significant results were obtained (all F ≤ 0.44; p ≥ 0.514). For the N2 ERP-component at electrodes CP3 and CP4, there was a significant main effect of “frequency” [F(1,22) = 35.33; p < 0.001; *η*_*p*_^*2*^ = 0.616] with a larger N2 amplitude in the 150 Hz (−6.4 µV/m² ± 0.9) than in the 40 Hz condition (−3.2 µV/m² ± 0.85). Further effects were insignificant (all F ≤ 0.54; p ≥ 0.472). The analysis of the N2 component at electrode Cz showed a main effect of “frequency” [F(1,22) = 15.45; p = 0.001; *η*_*p*_^*2*^ = 0.413] demonstrating a larger N2 amplitude in the 40 Hz (−4.4 µV/m² ± 0.92) than in the 150 Hz condition (−2 µV/m² ± 0.78). No other main or interaction effect was significant (all F ≤ 4.18; p ≥ 0.053).

### Residue iteration decomposition (RIDE)

**Figure 2:**
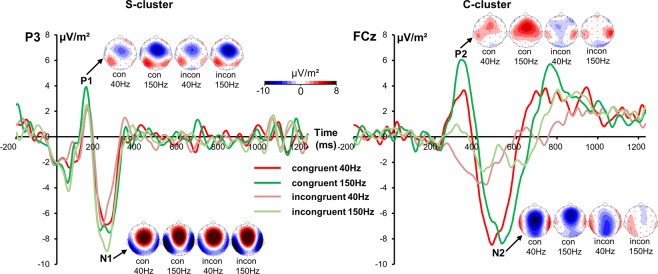
The P1 and N1 component in the S-cluster (left part of the figure) at electrode P3 and the P2 and N2 component in the C-cluster (right part) at electrode FCz is shown for congruent and incongruent trials in the 40 and 150 Hz conditions. Different colors of the electrophysiological time series represent 40 and 150 Hz and congruency conditions (congruent/incongruent) as shown in the legend. Time point 0 marks the stimulus presentation. The scalp topography plots show the S- and C-cluster at the peak of each cluster in 40 and 150 Hz congruent or incongruent trials. Red color indicates positive and blue negative values.

Neurophysiological data is shown in Fig. 2.

### S-cluster

The P1 ERP in the S-cluster quantified between 125 and 130 ms revealed in a main effect of “congruency” [F(1,22) = 5.21; p = 0.032; *η*_*p*_^*2*^ = 0.191] and a larger S-cluster amplitude was observed in congruent (2.3 µV/m² ± 0.73) than in incongruent trials (1.6 µV/m² ± 0.71). Other main or interaction effects were not significant (all F ≤ 3.94; p ≥ 0.060). For the N1 component a significant main effect of “frequency” was found [F(1,22) = 4.4; p = 0.048; *η*_*p*_^*2*^ = 0.167] showing that the N1 component was larger in the 150 Hz (−7.91 µV/m² ± 1.25) than in the 40 Hz condition (−6.54 µV/m² ± 1). No further main or interaction effects were significant (all F ≤ 3.03; p ≥ 0.096).

### C-cluster

The C-cluster was quantified at electrode FCz. Analysis of the positive deflection in the C-cluster revealed a main effect of “congruency” [F(1,22) = 24.38; p < 0.001; *η*_*p*_^*2*^ = 0.526] with a larger (i.e. positive) amplitude in the congruent (4.7 µV/m² ± 1.2) than in the incongruent condition (−0.8 µV/m² ± 1.4). Furthermore, the main effect of “frequency” was significant [F(1,22) = 5.33; p = 0.031; *η*_*p*_^*2*^ = 0.195]. It was shown that amplitudes were larger in the 150 Hz (3.2 µV/m² ± 1.53) than in the 40 Hz condition (0.6 µV/m² ± 1.16). The interaction effect was not significant [F(1,22) = 0.01; p = 0.946; *η*_*p*_^*2*^ < 0.001].

The negative deflection illustrated in the C-cluster was also analyzed. The main effect of “congruency” was significant [F(1,22) = 13.81; p = 0.001; *η*_*p*_^*2*^ = 0.386] and revealed larger amplitudes in congruent (−7.87 µV/m² ± 1.57) than in incongruent trials (−1.97 µV/m² ± 1.58). No further main or interaction effect was significant (all F ≤ 0.81; p ≥ 0.379).

### R-cluster

The R-cluster was quantified at electrodes C3 and C4 to register processes that take place above the motor cortices. Repeated measures ANOVA of the averaged signal showed no significant main or interaction effects (all F ≤ 0.86; p ≥ 0.365).

**Figure 3:**
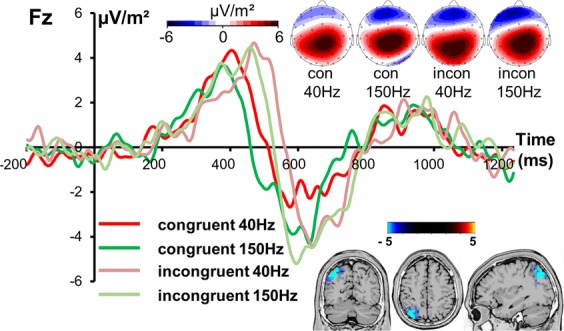
The R-cluster at electrode Fz is shown for congruent and incongruent trials in the 40 and 150 Hz conditions. Different colors of the electrophysiological time series represent SI- and SII-mediated processes and congruency conditions (congruent/incongruent) as shown in the legend. Time point 0 marks the stimulus presentation. The scalp topography plots represent the R-cluster in 40 and 150 Hz congruent or incongruent trials. Red color indicates positive and blue negative values. The sLORETA plots indicate the source of the difference in R-cluster amplitude modulations in congruent trials between 40 and 150 Hz conditions. The respective color scale presents critical t-values (corrected for multiple comparisons using SnPM).

The R-cluster showed the strongest signal at electrode Fz, as can be seen in Fig. 3. Repeated measures ANOVA demonstrated an interaction effect of “congruency x frequency” [F(1,22) = 4.68; p = 0.042; *η*_*p*_^*2*^ = 0.175] paralleling the interaction effect found in behavioral data. Post-hoc paired t-tests revealed that 40 Hz and 150 Hz conditions did not differ in incongruent [t(22) = 0.04; p = 0.969] but in congruent trials [t(22) = 2.52; p = 0.020]. In congruent trials, the R-cluster was larger (i.e. more negative) in the 150 Hz (−4.28 µV/m² ± 0.77) than in the 40 Hz condition (−1.48 µV/m² ± 1.08). Furthermore, congruent (−1.48 µV/m² ± 1.08) and incongruent trials (−4.39 µV/m² ± 1.02) significantly differed in the 40 Hz condition [t(22) = 2.64; p = 0.015] but not in the 150 Hz condition [t(22) = 0.12; p = 0.904].

## Discussion

In the current study, we examined the inter-relation of sensory processes and action control mechanisms with a focus on the processing modes during response selection; i.e. automatic vs. controlled selection. Underlying neurophysiological processes were investigated also considering the functional neuroanatomical level by means of source estimations. The study was based on considerations that somatosensory areas SI and SII differ in their ability to encode cognitive aspects of tactile processing^26^ relevant for behavioral decisions^27,66^. For this purpose, we created a tactile Simon task, because processing in the different experimental conditions is supposed to be mediated either via automatic or controlled processes. We made use of the fact that SI and SII are predominantly triggered by different frequency ranges^28^. The flutter sensation triggered by 40 Hz is assumed to mainly activate SI, whereas the 150 Hz vibration is expected to primarily evoke SII activation as outlined in the methods section.

In line with the hypotheses, the behavioral data revealed clear evidence for a differential effect of SI- (40 Hz) and SII- (150 Hz) mediated processes during response selection. It was shown that the IES score differed between SI-mediated and SII-mediated stimulus processing in congruent trials, which was not the case in incongruent trials. In congruent trials, the IES score was lower in the SII-mediated (150 Hz) condition. Since a smaller IES indicates more efficient (better) behavioral performance, response execution during congruent trials is more efficient when sensory information is processed via SII, compared to SI sensory areas. According to dual-process models, processing in congruent trials is mediated via an unconditional automatic (direct) route, while processing during incongruent trials is mediated via a conditional (controlled) route^10,13–17,19^. This shows that SI and SII cortical areas likely do not differentially affect performance when cognitive control is required during response selection. On a more general level, this suggests that sensory area specific processing differences (e.g. between SI and SII) only affect response selection when little cognitive control is required.

Neurophysiological data provide insight into mechanisms underlying behavioral effects. Performing analyses using standard ERP-components did not reveal any results paralleling behavioral effects. This is reasonable, because traditional ERPs represent a mixture of different signal sources like stimulus- and response related information^37,52,53,55,67,68^. The N2 ERP-component has been shown to represent intermingled stimulus-related perceptual processes and response-related motor processes^37,52,53^. The N2 and P3 time range is assumed to reflect processes like conflict monitoring and motor inhibition, respectively^69,70^. Yet, especially the N2/P3 time window yields intermingled processing codes originating from different sources of information^37,53,67^. As can be seen in standard ERP data (Fig. 1), the N2 component at electrode Cz likely reflects response selection processes rather than early perceptual processes due to the delayed latency roughly corresponding to C-cluster latency at electrode FCz (Fig. 2). Based on traditional ERPs, it would not be possible to make that observation. Yet, results reflecting behavioral data were not evident for standard ERPs. Notably, when accounting for different coding levels intermixed in neurophysiological data, the results show that experimental variations depending on sensory area-specific processing differences specifically affect motor processes – and not sensory ones. This is because only the R-cluster data, reflecting motor response-related processes like preparation or execution^34,54^, revealed interactive effects in line with the behavioral data. Although manipulating sensory processing, not the stimulus-related S-cluster but the response-related R-cluster reflecting response execution and preparation is modulated^54^. The R-cluster has already been associated with conflict and interference of information^52^. The R-cluster was larger (i.e., more negative) when processes were predominantly triggered via SII (150 Hz stimulation) compared to SI area (40 Hz stimulation) in congruent (non-conflicting) trials. A more negative R-cluster amplitude is hence associated with better behavioral performance. Notably, the source localization analyses show that these R-cluster modulations between the SI- (40 Hz) and SII- (150 Hz) mediated conditions were linked to activation differences in the superior parietal cortex (BA 7). Since this area is involved in integrating touch information and action^26,60,62^ fulfilling important sensorimotor functions^64,71^, it seems plausible that R-cluster modulations reflecting motor-related processes originate from superior parietal cortex. It is suggested that the posterior parietal cortex is involved in the transformation of tactile input into an external space since it has been shown that a disruption of this area using TMS impaired the localization of tactile information^62^. Superior parietal cortices have already been associated with tactile object localization^63^. Since the stimulus location is of crucial importance in the Simon task, it is plausible that the posterior parietal area is mutually involved. Furthermore, especially the left parietal cortex has been linked to movement control^72^, and the selection and preparation of motor effectors^73–78^. More precisely, it has been suggested that the parietal cortex codes the intended goal of movements and is therefore closely related to movement intention^64,65^. Referring to the behavioral data revealing better performance after SII- (150 Hz) triggered processes in congruent trials, the data suggest that parietal stimulus-response mapping processes are stronger using somatosensory stimuli that are preferentially processed in SII. This can be explained by the fact that the SII area has been shown to encode cognitive aspects of tactile processing^26,27,66^. In congruent trials, when no cognitive control has to be engaged, performance triggered via the SII area is superior because there is no conflict to be detected or resolved. Obviously, this enhances the specification of the movement goal in posterior parietal regions facilitating stimulus-response mapping. This is in line with the observation of stronger response preparation processes as reflected by the larger R-cluster amplitude. In congruent trials, it is therefore likely that responses are more efficiently prepared due to activation of the automatic processing route. In contrast, incongruent trials evoke a response conflict that requires cognitive control processes. Apparently, parietal motor goal specification processes are not sufficient to resolve this conflict. It is possible that frontal areas are additionally recruited like the medial frontal cortex and pre-supplementary motor area linked to response conflict^16,79,80^. This is plausible because SI, SII and the posterior parietal cortex are strongly linked to motor areas^26^ and somatosensory cortices also project to prefrontal areas^61^. A clear limitation of this study is the lack of direct evidence for predominant SI or SII activation by the different frequencies used. Since studies claim a tendency of stronger activation and not exclusive processing of one frequency in SI or SII, it would be beneficial to proof that the manipulation worked as expected. Nevertheless, it has already been shown that SI and SII differ in their efficacy to trigger sensorimotor integration processes to control responses^6^ and as results demonstrate, it is plausible to assume that there are differences in area-specific processing that are likely to unfold at the level of motor execution. Since there is no evidence of area-specific modulation of early (sensory) processes, it can be concluded that there is only a subtle effect of the predominance of one cortical area in the processing of certain frequencies. Furthermore, it is conceivable that an initial firing of both SI and SII^31,81^ in response to flutter and vibration is responsible that no effects on early ERPs can be found. However, it is possible that rather the difference in engaging in cognitive processing is the source of observed effects. Nonetheless, future studies should definitely use fMRI or TMS techniques to validate the differential involvement of somatosensory areas.

To summarize, we examined the inter-relation of sensory and response control processes during different processing modes (i.e. automatic vs. controlled selection). We demonstrated superior behavioral performance during congruent trials when sensory information is mediated via SII compared to SI cortical area. Based on temporally decomposed EEG signal we showed that experimental variations depending on sensory area-specific processing differences solely affect motor processes although sensory processes were manipulated. The modulation of motor-related processes was associated with activation differences in the superior parietal cortex (BA 7). We conclude that the SII cortical area supporting cognitive preprocessing of tactile input fosters automatic tactile information processing by facilitating stimulus-response mapping in posterior parietal regions.

## Materials and Methods

### Participants

We investigated N = 23 participants (14 females) in this study. Their age ranged between 18 and 30 years (mean age = 25; SEM = 0.82). Participants reported no psychiatric or neurological disorders and confirmed right-handedness. Subjects signed a written informed consent in accordance with the Helsinki Declaration of 1975, as revised in 2008. All methods were performed in accordance with the relevant guidelines and regulations. The study was approved by the institutional review board of the medical faculty of the TU Dresden.

### Procedure and task

**Figure 4:**
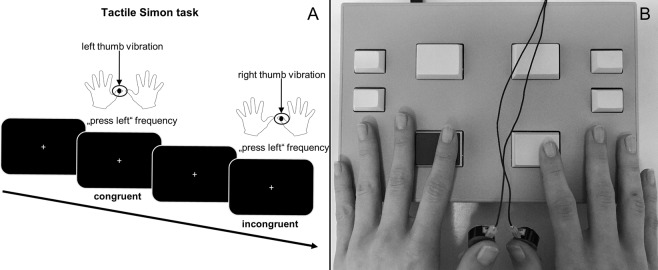
Schematic illustration of the task procedure (**A**) and the experimental set up (**B**). Participants were instructed to indicate stimulus frequency by pressing the left or right button but ignore the site of stimulation. Response and stimulation site were either congruent (corresponding sites) or incongruent (opposite sites). During the tactile Simon task, participants were asked to look at the white fixation cross on the computer screen.

To examine the differential effects of SI- and SII-mediated conflict control processes, a tactile version of the Simon task^7^ was conducted. The experimental paradigm is outlined in Fig. 4.

Small electromagnetic stimulators (Dancer Design; for more detailed information see http://www.dancerdesign.co.uk) were used to deliver vibro-tactile stimulation. A “main module” (Neurocore; http://www.neurocore.de/) was necessary to command the stimulators. The right and the left thumbs were used for stimulation. The thumbs were considered more suitable than other fingers since contact with the table can easily be prevented. To examine different conditions (i.e. SI- and SII-mediated processes), two different stimulation frequencies were used. Frequencies were chosen based on the observation that SI and SII cortical regions vary in their dominance to rather process low or high frequencies: whereas “flutter sensations” between 10 and 50 Hz are predominantly processed in the SI area, frequencies ranging from 100 to 400 Hz result in stronger activation of the SII cortex^28–31^. To trigger dominant SI activation, a 40 Hz vibro-tactile stimulation was used. This condition is therefore be referred to as “SI-mediated” condition. 150 Hz was applied to provoke dominant SII activation (“SII-mediated” condition). Those frequencies were selected since they are within above described ranges and are sufficiently distinct. Stimulation duration was set to 100 ms since it was shown to be sufficient for stimulus categorization^6^. Prior to the experiment participants were familiarized with the stimuli as a part of a short exercise to ensure the ability to categorize the stimuli correctly. A white fixation cross was shown in the middle of a black screen. Participants were required to respond as fast and accurate as possible to stimulus presentation by pressing either the left or the right response button (the mapping of response side to stimulus type changed between the blocks) with their right or left index fingers regardless of whether the right or the left stimulator was vibrating. Hence, participants were told to ignore stimulation side and just respond to the type of stimulation (high or low). Prior to each block, participants were informed which type of frequency (high or low) was assigned to which response side (left or right button press). Thus, trials were either congruent (i.e. stimulation side corresponded to the response side) or incongruent (i.e. when stimulation and response sides were opposite). In total, 832 trials subdivided into 4 blocks were presented to the participants. The mapping of response side and stimulus type was blocked. The trial sequence within the blocks was pseudo-randomized. The inter-trial-interval was jittered between 700 and 1100 ms so that stimulus onset was not predictable. In case the button press occurred within a time range from 100 to 1000 ms after stimulus presentation, the answer was classified as correct.

### EEG recordings and analysis

To record the EEG, 60 passive Ag/AgCl ring electrodes at equidistant positions connected to a QuickAmp amplifier (BrainProducts Inc.) were applied. The position of the ground and reference electrodes was at coordinates theta = 58, phi = 78 and theta = 90, phi = 90, respectively. Electrode impedances were below 5 kΩ. Sampling rate was set to 500 Hz and recording bandwidth ranged from 0.5 to 80 Hz. A down-sampling to 256 Hz was conducted after data were recorded. By means of an IIR band-pass filter, the EEG data was filtered from 0.5 to 20 Hz with a slope of 48 db/oct and a 50 Hz notch filter. Afterwards, an initial manual raw data inspection was performed to eliminate infrequent (technical and muscular) artifacts. On that basis, an independent component analysis (ICA; infomax algorithm) was run on the data for all blocks combined to reveal artifacts related to eye movements like blinks or lateral movements. ICA components clearly representing artifacts were excluded manually. As preprocessing was completed, segmentation was performed separately for the 40 and 150 Hz conditions in congruent and incongruent trials. A segment comprised the period of −200 ms before stimulus presentation to 1200 ms after it. Only trials with correct responses were included in the data analysis, which means that the correct response button had to be pressed within 100 to 1000 ms after stimulus presentation. Afterwards, an automated artifact rejection was run to reject trials with a maximal value difference of 200 µV in a 200 ms period. Further rejection criteria were amplitudes below −200 µV and above 200 µV as well as amplitudes below 0.5 µV in a 100 ms interval. In the following step, current source density (CSD) transformation was performed using four splines and ten polynomials to remove reference potential and re-reference the data leading to amplitude values in µV/m². CSD is applied since it acts as a spatial filter^82,83^, which makes it easier to identify electrode sites that best reflect relevant neuronal activity. This was followed by a baseline correction from −200 to 0 (0 represents the time of stimulus presentation). Subsequently, different conditions (congruent/incongruent and 40/150 Hz stimulations) were averaged on single-subject level. Scalp topography plots were used as a basis to choose relevant electrodes sites for data quantification. This revealed electrodes P7 and P8 as most suitable to examine P1 and N1 ERP-components. For electrode P7, the time window from 125 to 160 ms was used for quantification of all conditions. The N1 component was calculated on the basis of the time range between 180 to 280 ms. At electrode P8, P1 ERP-component was quantified in the range from 140 to 170 ms and the N1 ERP-component between 180 to 300 ms for all conditions. The N2 ERP-component was quantified at electrodes CP3 and CP4, as well as electrode Cz. The time range of quantification for electrode CP3 was 155 to 285 ms and for electrode CP4 145 to 260 ms across different conditions. At electrode Cz, the time window from 310 to 450 ms was used for SI- and SII-mediated incongruent conditions and the interval between 360 to 480 for SI- and SII-mediated congruent conditions. This choice of electrodes and time intervals used to quantify the ERP-components was validated by a statistical procedure described in Mückschel *et al*.^84^, which revealed the same electrodes as previously chosen by visual inspection. During the statistical procedure, the amplitude of the ERP is extracted at all 60 electrode sites in each of the mentioned search intervals. To compare each electrode against the average of all other electrodes, Bonferroni-correction was applied for multiple comparisons (critical threshold p = 0.0007). Only the electrodes solely exhibiting significantly larger mean amplitudes (i.e., negative for N-potentials and positive for the P-potentials) compared to the remaining electrodes were chosen.

### Residue iteration decomposition (RIDE)

As done in prior work^52,54,58^, the RIDE toolbox (available on http://cns.hkbu.edu.hk/RIDE.htm) was used to perform RIDE analysis using MATLAB (MATLAB 12.0; Mathworks Inc.). Mathematical details of this method can be found in Ouyang *et al*.^55^. In principle, the RIDE algorithm decomposes ERP-components through the use of L1-norm minimization procedure (i.e. producing median waveforms). As a result, the residual error arising from temporal variability in single trials is minimized^55^. RIDE decomposition works on the basis of latency variability and is performed separately for each electrode without reference to scalp distributions or waveforms^34^. Consequently, results are not biased by the previously implemented CSD transformation. The RIDE algorithm parses the ERP signal into clusters either related to stimulus onset (S-cluster) or response time (R-cluster). Furthermore, a C-cluster with variable latency, temporally located between stimulus and response is extracted by initially estimating and then iteratively improving the waveform. This is implemented by a self-optimized iteration scheme ameliorating C-cluster latency estimation. To obtain a starting point, a time window function estimates the initial C-cluster latency. Then, an iteration processes is initiated in which the S-cluster is removed and the C-cluster latency re-estimated utilizing a template-matching approach. This is carried out until initial latency estimation and S- and C-cluster converge. In case of the current study, the time window used to start with C-cluster estimation has been set to 200 to 800 ms after stimulus onset. A time window confinement is performed for each cluster. The time windows are defined in such a way that they cover the range in which the clusters are supposed to occur. Consequently, values have to be adjusted to fit the data on application^55^. The S-cluster was expected to occur in the time range between −200 to 400 ms around stimulus onset and the R-cluster in the time window from −300 to 300 around average response times. Please refer to Ouyang *et al*.^34,54,55^ for deeper insights into the method and mathematical details.

Electrodes were selected as described in the standard ERP-component section. The S-cluster was quantified at electrode P3 in the time range between 125 and 130 ms to measure the P1 component. Furthermore, the N1 component in the S-cluster was quantified between 220 and 240 ms. The R-cluster was quantified at electrode Fz in the time window from 585 to 590 in the congruent 40 Hz condition, from 610 to 615 ms in the congruent 150 Hz condition, from 620 to 625 ms in the incongruent 40 Hz and from 620 to 630 in the incongruent 150 Hz condition. To take motor processes into account, the electrodes C3 and C4 were quantified and averaged afterwards. For both electrodes corresponding time windows were chosen. Time intervals were determined based on mean reaction times in different conditions: for the congruent 40 Hz condition, quantification based on the time range from 463 to 503 ms, for the congruent 150 Hz condition it ranged from 450 to 490 ms and the time window for the incongruent 40 and 150 Hz conditions was set to 500 to 540 ms. The C-cluster was quantified at electrode FCz. The positive peak (i.e. the P2 component) was calculated based on the time window from 300 to 330 ms for all conditions. The negative deflection (i.e. the N2 component) in the C-cluster was quantified in the time interval from 440 to 460 ms in the 40 Hz congruent and incongruent conditions and the time period from 490 to 510 ms was used for the 150 Hz congruent and incongruent conditions. The choice of electrodes and time windows was done as performed for the ERP data (see above).

### Source localization analysis

Source localization analysis was performed using sLORETA (standardized low resolution brain electromagnetic tomography)^33^ and was based on RIDE data. sLORETA provides a single solution to the inverse problem^33,85,86^. In principle, the intracerebral volume is split into 6239 voxels with a spatial resolution of 5 mm. Afterwards, standardized current density calculation is performed at each voxel in a realistic head model^87^ resting on MNI152 template^88^. sLORETA produces reliable results without localization bias which has been verified mathematically^86^. More validation of sources identified by sLORETA has been provided by EEG/fMRI and neuronavigated EEG/TMS studies^86,89^. The sLORETA-built-in voxel-wise randomization tests with 2500 permutations which is based on statistical nonparametric mapping (SnPM) was utilized to compare the voxel-based sLORETA images across conditions; i.e. 40 and 150 Hz conditions were contrasted. Afterwards, voxels exhibiting significant differences (p < 0.01, corrected for multiple comparisons) between calculated contrasts were transferred to the MNI brain.

### Statistical analysis

Behavioral and neurophysiological data were analyzed using repeated measures ANOVAs (percentage of hits and hit reaction times in 40 and 150 Hz stimulation congruent and incongruent trials). Within-subject factors included in analyses were “congruency” (congruent/incongruent) and “frequency” (40/150 Hz conditions). All tests were Greenhouse–Geisser corrected and all post hoc tests Bonferroni corrected. For descriptive statistics mean values and the standard error of the mean (SEM) are presented in brackets in the results section.

## Data Availability

The datasets generated during and/or analyzed during the current study are available from the corresponding author on reasonable request.
